# Development and validation of a population based risk algorithm for obesity: The Obesity Population Risk Tool (OPoRT)

**DOI:** 10.1371/journal.pone.0191169

**Published:** 2018-01-18

**Authors:** Michael Lebenbaum, Osvaldo Espin-Garcia, Yi Li, Laura C. Rosella

**Affiliations:** 1 Dalla Lana School of Public Health, University of Toronto, Toronto, Ontario, Canada; 2 Institute for Clinical Evaluative Sciences, Toronto, Ontario, Canada; 3 Public Health Ontario, Toronto, Ontario, Canada; Dana-Farber Cancer Institute, UNITED STATES

## Abstract

**Background:**

Given the dramatic rise in the prevalence of obesity, greater focus on prevention is necessary. We sought to develop and validate a population risk tool for obesity to inform prevention efforts.

**Methods:**

We developed the Obesity Population Risk Tool (OPoRT) using the longitudinal National Population Health Survey and sex-specific Generalized Estimating Equations to predict the 10-year risk of obesity among adults 18 and older. The model was validated using a bootstrap approach accounting for the survey design. Model performance was measured by the Brier statistic, discrimination was measured by the C-statistic, and calibration was assessed using the Hosmer-Lemeshow Goodness of Fit Chi Square (HL χ^2^).

**Results:**

Predictive factors included baseline body mass index, age, time and their interactions, smoking status, living arrangements, education, alcohol consumption, physical activity, and ethnicity. OPoRT showed good performance for males and females (Brier 0.118 and 0.095, respectively), excellent discrimination (C statistic ≥ 0.89) and achieved calibration (HL χ^2^ <20).

**Conclusion:**

OPoRT is a valid and reliable algorithm that can be applied to routinely collected survey data to estimate the risk of obesity and identify groups at increased risk of obesity. These results can guide prevention efforts aimed at reducing the population burden of obesity.

## Introduction

The prevalence of obesity has risen dramatically across the globe and has become a major global challenge for public health officials across the developed and developing world [1]. Given the rising prevalence of obesity and obesity-related conditions, such as diabetes, cardiovascular disease, and osteoarthritis, obesity poses a significant burden on individuals and health care systems [2]. Studies have demonstrated significant increases in physician, drug, hospital, and total health care costs with total estimates attributing to obesity tens of billions in total costs each year in the United States [3].

Assessing the future burden of diseases and how risk factors may affect this burden is key in informing population health efforts [4]. Although numerous studies have applied various methods to forecast the future burden of obesity, such as linear or nonlinear extrapolations of prevalence or simulation methods, the use of risk prediction models to estimate the absolute risk of obesity or weight related outcomes is uncommon [5–8]. With other conditions, such as with cardiovascular disease, clinical risk prediction models such as the Framingham Risk Score have long been used to characterize risk and inform both prevention and treatment [9,10].

Traditionally risk prediction models have typically relied on variables available in the clinic that are not routinely collected at the population level or only collected in surveys with small samples and limited. This has limited their use for the purpose of surveillance and health planning, which usually takes place at lower geographic levels such as the state/province or sub-region.

Population risk tools represent an alternative approach to risk prediction and forecasting of the future burden of disease. Models built with this approach use samples derived from and variables limited to population surveys to develop risk prediction models, which are then validated within a traditional risk prediction framework. These models can then be applied to routine population surveys to forecast the burden of disease at the sub-regional, regional and national level [4]. Rosella et al. previously developed the Diabetes Population Risk Tool (DPoRT), a population risk tool that predicts the future risk of diabetes and has been used by public health planners at the regional and provincial level to forecast the future burden of diabetes under different scenarios to inform public health policies [11–12]. Subsequent models for stroke and all-cause mortality have also been developed [13,14] To our knowledge, no obesity projection studies have used a risk model approach that work on population survey data. The objective of this study was to develop a population risk tool for obesity, the Obesity Population Risk Tool (OPoRT), which can be widely used to estimate the future burden of obesity, to identify subgroups in the population at elevated risk, and provide a tool that can be used to inform obesity prevention.

## Materials and methods

### Data

We used the National Population Health Survey (NPHS), a nationally representative longitudinal survey of Canadians of all ages that started in 1994–5 and followed 17,626 individuals of all ages every two years until 2010–11, for a total of nine cycles. The NPHS sampled household residents from the ten provinces and excluded respondents who resided in the territories, were institutionalized, resided on aboriginal reserves or crown lands, or who were full time members of the Canadian forces living on Canadian forces bases. Respondents were sampled through stratified clustered sampling. Further details on the sample design and methodology of the NPHS can be found elsewhere [15,16]. Given the change in interview method from in-person to telephone interview following the initial cycle (1994–5), we used the second cycle (1996–7) as our baseline. This resulted in 14 years of follow-up until 2010–11. We excluded individuals less than 18 years or greater than 99 years of age at baseline, respondents with extreme body mass index (BMI) (BMI < 10 or BMI > 70 kg/m^2^), those who were pregnant or missing BMI at baseline, and those with missing baseline data on model covariates.

### Analysis

We design the model so it can be applied to routinely collected population surveys and thus only variables that were available at baseline and consistently collected in population surveys were included. We assessed the inclusion of time, baseline BMI, age, marital status, living arrangements, smoking status, leisure time physical activity, education, equivalized income quintiles (income adjusted for household size), ethnicity, immigration status, rural status, alcohol consumption, and household ownership. Both baseline BMI and age were tested in continuous and categorical specifications and interactions were assessed between time, baseline BMI, and age. Given the bias in self-reported BMI, we applied a validated BMI correction equation to all analyses [17]. We added variables sequentially, controlling for variables already in the model, based on importance as determined by prior studies and marginal predictive (Brier Score, C statistic, and Hosmer Lemeshow Goodness of Fit Chi Square (HL χ^2^)) and statistical significance.

Given there were seven follow-up cycles, model development was conducted using logistic regression with generalized estimating equations (GEE) with the GENMOD procedure in SAS (Version 9.3). These models appropriately model longitudinal binary data by using a correlation matrix to account for the clustering of observations within individuals across time [18]. Furthermore, given that the data sets OPoRT will be applied to only have baseline data, a marginal model (i.e. GEE) is preferred over a mixed model approach. We assessed the appropriateness of a number of correlation matrices including independence, unstructured, autoregressive (AR) (1), and exchangeable. Given the conceptual appropriateness and reasonable calibration, AR(1) correlation matrices were selected. Since GEEs are marginal models, predictions correspond to the average estimated risk for a given individual with specific characteristics.

### Validation

We validated our data internally using data from 10 years follow-up (2005–6, NPHS Cycle 7) using standard validation criteria for the development of risk prediction models. We assessed the overall performance of the model with the Brier score, a measure equal to the sum of the square deviation of the prediction from the observed value divided by the total sample size [19]. It ranges from 0–1, with 0 representing perfect predictions and a value of 0.33 or greater indicating random predictive ability [19]. We multiplied the squared deviations by the survey weight and divided this by the total of the sample weights to estimate a survey weighted Brier score. Given, the value of the Brier score is dependent on the prevalence of the outcome, we the scaled Brier score by its maximum value so that values ranged from 0–1[19]. We assessed calibration using the Hosmer Lemeshow Goodness of Fit Chi Square (HL χ^2^ test) [19,20]. The HL χ^2^ test is a measure of the overall fit of a statistical model which compares agreement between observed and predicted risk across deciles of predicted risk [19]. Consistent with other risk prediction models, including the Framingham risk score, we used a HL χ^2^ value < 20 (P < 0 .01) to represent sufficient calibration [12]. We assessed discrimination with the c-statistic, which measures the ability of the model to rank order individuals from low to high risk. The values of the c-statistic are equivalent to the area under the receiver operating characteristic curve, and represent the probability that a randomly chosen case has a greater predicted risk than a randomly chosen non-case [19]. Values of 0.5 represent random predictions, while values between 0.7 and 0.8 indicate reasonable discrimination, and values of 0.8 and greater represent excellent discrimination [20,21]. Calibration curves plotting the smoothed relationship between predicted and observed risks were created using the RMS (Regression Modeling Strategies) package in R [22]. We also examined the validation characteristics of the final model in individuals not obese at baseline.

Due to a lack of external data to validate the individual longitudinal predictions, we conducted an internal validation using the 0.632+ Bootstrap method to assess the potential optimism of the model [23]. However, this method was not originally intended for complex longitudinal survey data. Consequently, extensions of this approach were proposed and have been developed into a SAS macro by the research team. Briefly, the complex survey nature of the data was considered in the bootstrap re-sampling scheme by selecting individuals into the bootstrap samples by probability proportional to size sampling with replacement, which used the inverse of each individuals’ sampling weight as the probability of selecting them into the bootstrap samples.

To ensure the model was representative of the Canadian population, we applied longitudinal survey weights. These weights also take into account non-response rates at baseline and follow-up. Survey weights were applied in model development, in the calculation of the Brier score, and in the bootstrap validation. All analyses including descriptive analyses, model development and bootstrap validation were sex stratified.

## Results

The prevalence of obesity in the cohort grew from 18.4% to 28.7% from baseline to 10 years for males and from 17.6% to 26.6% for females. The predicted risk of obesity at this time point is 29.0% and 26.2% for males and females respectively. The sex-specific mean and standard error or prevalence of all predictors can be found in Table 1.

**Table 1:** Descriptive characteristics of the cohort at baseline.

	Male	Female
Variables	N = 5474	N = 6402
Body Mass Index	26.9 (0.07)	25.9 (0.08)
Age	43.6 (0.28)	45.5 (0.28)
Obese	18.4	17.6
Former smoker	36.0	28.3
Current smoker	32.1	26.8
Live with a spouse/partner	24.9	23.3
Parent living with a spouse/partner and children	38.1	34.0
Single parent living with children	1.4	8.0
Other living arrangement	22.6	19.2
Any post-secondary education	62.5	
Non drinker	14.9	
Physically Inactive		61.1
Asian		2.9
Aboriginal		0.5
South Asian		2.4
Black		1.8
Other Ethnicity		1.5

Categorical variables are presented as proportion (%), while continuous variables are presented as mean (SE).

The sex specific OPoRT functions for males and females are presented in Table 2. The strongest predictors of obesity were measures of baseline BMI, baseline age, obesity, and time. Both equations include all of these variables and interactions among them. Continuous BMI and age were found to result in the greatest model performance as assessed by brier score and c-statistic. Smoking status and living arrangements were found to be important predictors for both sex. Sex-specific variables include any post-secondary education and non-drinking status for males and physical inactivity and ethnicity for females.

**Table 2:** Obesity Population Risk Tool (OPoRT) function for males and females.

Males			Females		
Predictors	Coefficients	P value	Predictors	Coefficients	P value
Intercept	7.7187	0.386	Intercept	-18.0283	0.0002
Time	0.5603	< .0001	Time	0.5214	< .0001
Time^2^	-0.0225	0.0002	Time^2^	-0.0169	0.0047
BMI	-1.4073	0.0341	BMI	0.6156	0.101
BMI^2^	0.0392	0.0015	BMI^2^	0.0001	0.9884
Age	-0.0208	< .0001	Age	-0.0223	< .0001
Age X Time	-0.005	< .0001	Age X Time	-0.0038	< .0001
Obese X BMI	0.6968	< .0001	Obese X BMI	0.2808	0.0061
Obese X BMI^2^	-0.0234	< .0001	Obese X BMI^2^	-0.0093	0.0059
Obese X Age	0.0203	0.0069	Obese X Age	0.0216	0.0019
Obese X Age X Time	-0.0043	< .0001	Obese X Age X Time	-0.0048	< .0001
Former smoker	0.2979	0.0024	Former smoker	0.0538	0.5668
Current smoker	0.3182	0.0024	Current smoker	0.2463	0.028
Live with a spouse/partner	-0.2332	0.0714	Live with a spouse/partner	-0.1879	0.115
Parent living with a spouse/partner and children	-0.1758	0.1433	Parent living with a spouse/partner and children	-0.2145	0.0969
Single parent living with children	0.4612	0.1628	Single parent living with children	-0.2659	0.1291
Other living arrangement	-0.0497	0.7483	Other living arrangement	0.0885	0.5789
Any post-secondary education	-0.1472	0.0877	Physically inactive	0.233	0.0061
Non-drinker	0.2336	0.0591	Asian	-1.2186	0.0096
			Aboriginal	0.5767	0.3065
			South Asian	-0.4955	0.0901
			Other	0.4868	0.2198
			Black	0.2534	0.343

Reference groups: never smoker, unattached individual living alone, white ethnicity.

**Fig 1:**
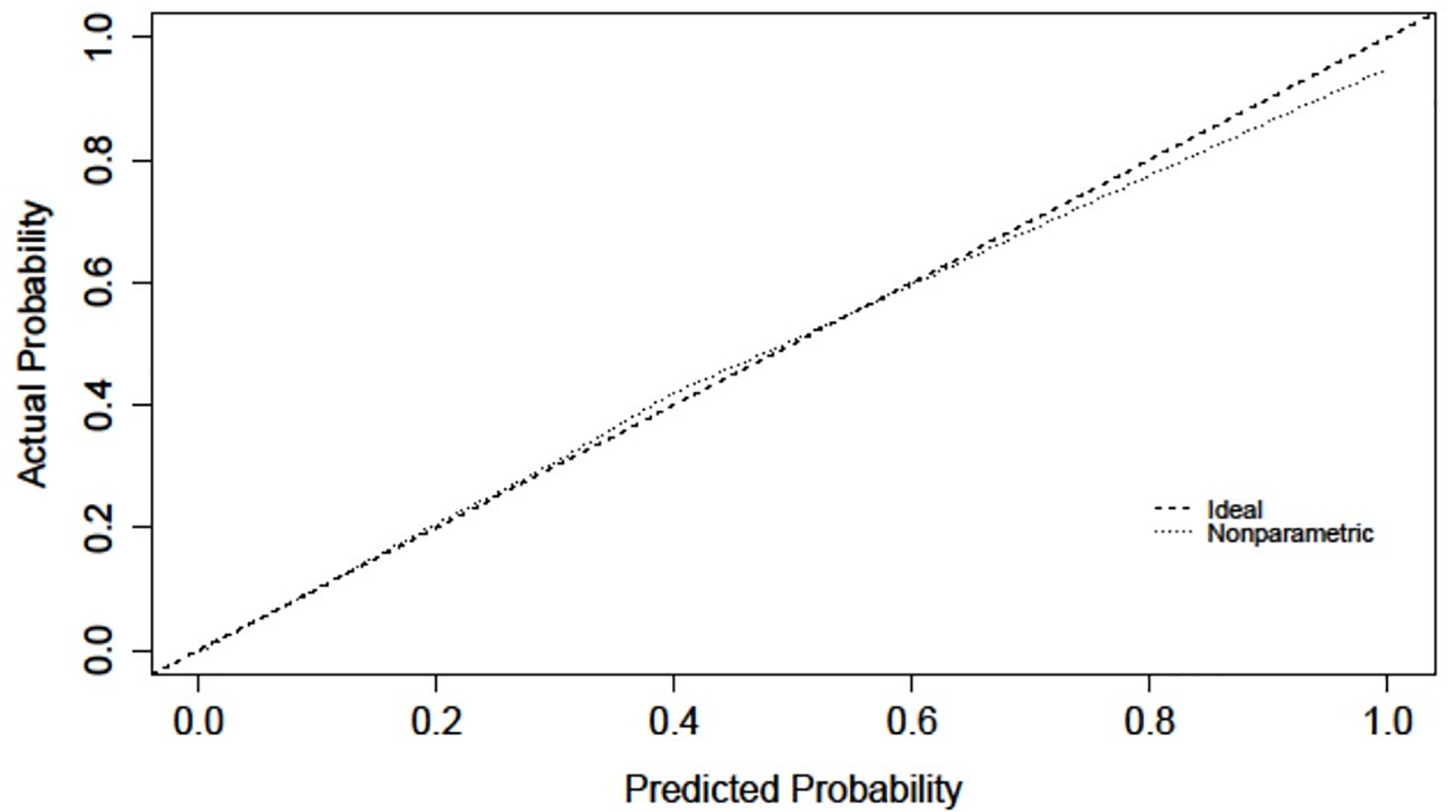
Calibration plot demonstrating the relationship between predicted and observed risk among males.

**Fig 2:**
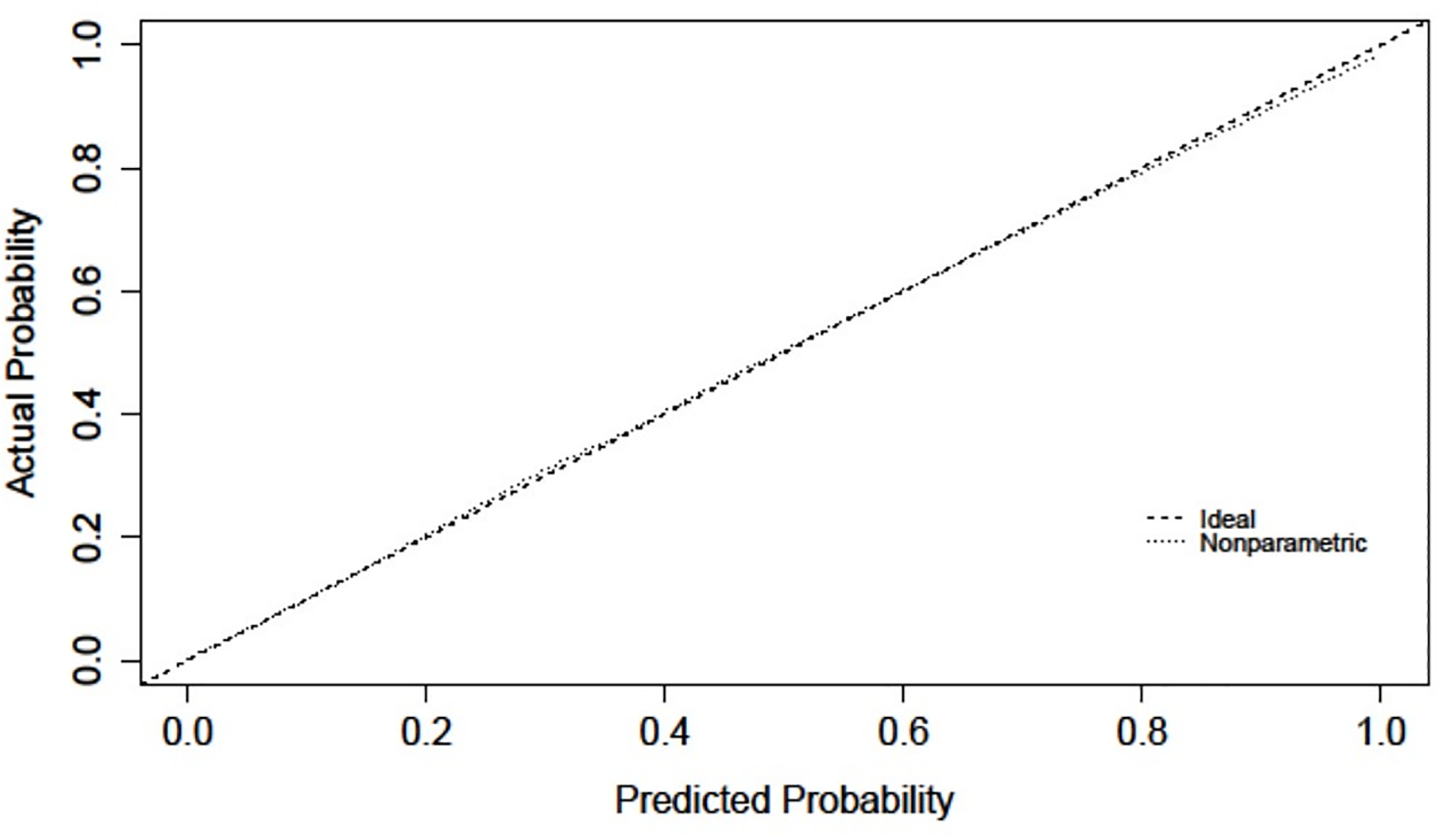
Calibration plot demonstrating the relationship between predicted and observed risk among females.

The validation characteristics for each model are presented in Table 3. The overall performance of both models as measured by the Brier score is good and the calibration of the models as measured by the Hosmer-Lemeshow Chi Square (HL χ^2^) is acceptable. Meanwhile, the discrimination as measured by the c-statistic of both models is excellent. Models for both sex performed similarly well in the bootstrap validation, with optimism corrected brier statistic equalling 0.118 for males and 0.095 for females. Given restrictions on the release of small cell sizes prohibited the release of plots of deciles of predicted versus observed risk, calibration plots were used to demonstrate the smoothed relationship between predicted and observed risk. As shown in Figs 1 and 2, calibration plots also demonstrated excellent calibration with close agreement between predicted and observed risks for both males and females.

**Table 3:** Obesity Population Risk Tool (OPoRT) Internal validation characteristics for males and females.

	Males	Females
Brier Score (Scaled)	0.115 (0.44)	0.090 (0.535)
C-Stat	0.890	0.918
HL χ^2^	15.064	12.210
HL χ^2^ p-value	0.058	0.142

For males, restricting the validation of the model to individuals not obese at baseline resulted in the Brier score (scaled brier) decreasing in performance to 0.108 (0.251) and c-statistic decreasing in value to 0.837, and calibration remained acceptable (HL χ^2^ = 5.98, p = 0.649). For females, restricting validation to individuals not obese at baseline resulted in the Brier score (scaled brier) decreasing in performance to 0.083 (0.291) and c-statistic decreasing in value to 0.85, and calibration remained acceptable (HL χ^2^ = 13.23, p = 0.104). A sample calculation showing how 10 year (i.e. 5 cycles) risk is estimated are included in S1 Text.

## Discussion

This study provides the first example of a population risk prediction tool that can be applied to estimate the future risk of obesity based on multiple risk factors regularly available in population health surveys. The Obesity Population Risk Tool (OPoRT) was found to be discriminating and demonstrated good overall performance and calibration, with high agreement between observed and predicted values. This tool represents a novel approach to assessing obesity risk that can be used to inform public health policy on obesity prevention.

Despite using variables limited to population surveys, the Brier score of the model demonstrated reasonable performance in the internal validation. Results of internal validations in developmental data are often referred to as apparent model validity, which may be optimistic given they are validated in the same data source used to build the model [24]. Given this we conducted a bootstrap validation which demonstrated a similar optimism corrected Brier scores. The model had high discriminatory likely due to the strong association between baseline BMI and future obesity. Risk prediction models for other conditions including hypertension and diabetes also demonstrate similarly high discrimination given they commonly include similar baseline measures related to the condition such as blood pressure for hypertension or fasting plasma glucose for diabetes [25,26].

Risk prediction models typically model disease incidence and individuals with the condition at baseline are excluded from the development cohort. However, we included individuals with obesity at baseline given that it is possible for individuals with obesity to revert to non-obesity and the intended application of the model for forecasting prevalence. Although overall performance and discrimination was lower, the model maintained excellent discrimination and acceptable calibration in both males and females among adults not obese at baseline.

A greater focus on obesity prevention is necessary given the large disease burden due to obesity and the limited long-term effectiveness of most weight loss interventions [27]. To date, there has been limited response and slow implementation of population based policies [28]. Although prioritized action among certain population subgroups will be important for prevention, it is uncommon for prevention studies to select individuals based on their risk for obesity [28]. As such the tool developed in this study can be used to inform the prioritization of individuals for intervention based on multiple risk factors. This is the suggested practice in the prevention of cardiovascular disease, with clinical guidelines recommending the use of risk algorithms [29]. Similarly, the DPoRT model has been used by public health planners to inform prevention in their jurisdiction by estimating the absolute risk of diabetes, identifying populations at greatest risk, and estimating the effects of different proposed interventions [12,30]. There have also been health promotion applications of population risk tools, with a mortality model being incorporated into an online life expectancy calculator used by thousands of individuals to inform individuals how their lifestyle risk factors may be influencing their life expectancy [14]. OPoRT may be able take on similar roles with obesity prevention.

To the knowledge of the authors, this is the first risk prediction model that has been developed for the purpose of obesity forecasting, and one of the few risk prediction models that have been developed for obesity or weight related outcomes. A previous risk prediction model was developed for substantial weight gain (SWG) over 5 years in the European Prospective Investigation into Cancer and Nutrition (EPIC) cohort [9]. Despite predicting a different weight outcome, OPoRT and the model for SWG have a number of common predictors including age, baseline weight, education, smoking status, exercise and alcohol consumption [9]. Both of these studies suggest there are numerous risk factors beyond current weight that are important for predicting future weight outcomes. The SWG model also included dietary predictors including consumption of red and processed meat, bread, and soft drinks. Given reproductive factors were not available and dietary variables were unavailable until later NPHS cycles, neither were included in the OPoRT model. However, given the high discrimination, only addition of very strong risk factors would further improve discrimination [31]. Despite the availability of dietary variables, the SWG risk prediction model only achieved modest discrimination (c = 0.64) [9], suggesting that although dietary variables may be associated with weight outcomes, they may be of limited use for prediction.

Other approaches to forecasting the future burden of obesity have included fitting linear or non-linear time trends to repeat cross-sectional data [5,7,8], the use of simulation methods [6], and projection of obesity prevalence using a cohort [32]. A limitation of these past approaches has been the exclusive focus at the population level and these studies generally have not assessed the impact of individual risk factors on future obesity prevalence and did not assess specific high-risk populations which can inform targeted obesity-related interventions and thus limit their application for informing prevention strategies.

A strength of this study was the use of 14 years of data over several collection periods enabling appropriate modeling of long-term trends even in the presence of fluctuations in body weight over time. In addition, this study used a nationally and provincially representative cohort, which maintained high response rates throughout the study period, with a cycle 9 response rate of 69.7% [33]. We also used vigorous validation metrics to ensure the model is accurate for population health planning purposes as well as has wide applicability by being able to run population health surveys with variables that are widely collected globally.

This study also has limitations that should be taken into consideration. Given administrative codes greatly underestimate obesity prevalence and there are no Canadian representative population based studies with repeated assessment of measured height and weight, this study relied on self-reported height and weight, which underestimates BMI and the prevalence of obesity [34]. Although this may affect the calibration of the model, it is unlikely to have affected the discrimination as demonstrated by a previous simulation study [35]. We attempted to reduce this bias by applying BMI correction equations derived from the 2005 CCHS [17], a survey which was based on and later replaced the NPHS. Second, there was a lack of data for external validation of the model. However, most risk prediction algorithms are initially published without external validations [24] and we used bootstrap validation, the optimal internal validation technique [36]. OPoRT should be independently assessed in samples from other countries although the model re-calibration may be necessary given differences in risk across populations and time [37]. Third, although individuals with a baseline BMI value and at least one follow-up response were included in the model, our study excluded follow-up points where individuals were deceased or dropped out. Given this is unlikely to be missing completely at random, some bias in our models estimates may have been introduced [38]. Given it is unclear how bootstrap resampling should be applied with multiple imputation [39], future work should explore the use of longitudinal multistate prediction models that can additionally model death and drop-out.

### Conclusion

The Obesity Population Risk Tool (OPoRT) represents a novel, valid and accurate risk prediction model for obesity. OPoRT demonstrates both good calibration and excellent discrimination for the prediction of obesity at the population. Given that obesity is a key global contributor to the burden of chronic disease, combating the obesity epidemic has become a high government priority and prevention is urgently needed. By characterizing the risk and distribution of risk among a population, population risk tools including OPoRT can be used to inform health planning including the prioritization of groups for prevention.

**Ethics:** This study received ethical approval from University of Toronto Research Ethics Board (Protocol #28094)

## Supporting information

S1 Text Sample calculation of obesity risk using the Obesity Population Risk Tool (OPoRT). (PDF)
