# Supplementary material for: Development and validation of a population based risk algorithm for obesity: The Obesity Population Risk Tool (OPoRT)
Source: PLoS One. 2018 Jan 18;13(1):e0191169. doi: 10.1371/journal.pone.0191169 (PMC5773177; doi:10.1371/journal.pone.0191169)
Supplement: S1 Text — (PDF) [file pone.0191169.s001.pdf]

S1 Text. Sample calculation of obesity risk using the Obesity Population Risk Tool (OPoRT)

Estimate the log odds:

Log odds = 7.7187 + (time[&] *0.5603) + ($time^2$*-0.0255) + (BMI*-1.4073) + (BMI^2 *0.0392) + (Age*-0.0208) + (Age * Time*-0.005) + (Obese*BMI*0.6968) + (Obese* BMI^2*-0.0234) + (Obese*Age*0.0203) + (Obese*Age*Time*-0. 0043) + (Former Smoker*0.2979) + (Current Smoker*0.3182) + (Live with a spouse/partner *-0.2332) + (Parent living with a spouse/partner and children*-0.1758) + (Single parent living with children*0.4612) + (Other living arrangement*-0.0497) + (Any post-secondary education*-0.1472) + (Non-drinker*0.2336)

= 7.7187 + (5 * 0.5603) + (5^2 * -0.0255) + (25 *-1.4073) + (25^2 *0.0392) + (40 * - 0.0208) + (40 * 5 * -0.005) + (0 * 25 * 0.6968) + (0 * 25^2 *-0.0234) + (0 * 40 * 0.0203) + (0 * 40 * 5 *-0. 0043) + (0 * 0.2979) + (1 * 0.3182) + (1 * -0.2332) + (0 * -0.1758) + (0 *0.4612) + (0 *-0.0497) + (1*-0.1472) + (0*0.2336)

= -2.619

1. Estimate the odds:

Odds = exp (-2.619) = 0.0729

2. Estimate the risk:

Risk = odds / (1 + odds)

= 0.0729 / (1 + 0.0729)

= 0.0679

[&]Time is measured in 2 year intervals (i.e. 10 years = 5).
